# A robust bitmap-based real-time position tracking algorithm for rats in radial arm maze tests

**DOI:** 10.1038/s41598-021-01974-0

**Published:** 2021-11-17

**Authors:** Chi-Chun Chen, Liang-Jyun Hong, Jian-Yong Wang, Ching-Ping Chang

**Affiliations:** 1 Department of Electronic Engineering, National Chin-Yi University of Technology, Taichung, Taiwan; 2 Department of Medical Research, Chi Mei Medical Center, Tainan, Taiwan

**Keywords:** Biomedical engineering, Electrical and electronic engineering, Spatial memory

## Abstract

This paper aims to develop a position tracking algorithm by which a rat in a radial arm maze can be accurately located in real time. An infrared (IR) night-vision camera was hung above the maze to capture IR images of the rat. The IR images were binarized and then duplicated for subsequent intersection and opening operations. Due to simple operations and a high robustness against the noise spots formed by the droppings of the rat, it took just minutes to process more than 9000 frames, and an accuracy above 99% was reached as well. The maze was intruded by an experimenter to further test the robustness, and the accuracy slightly fell to 98%. For comparison purposes, the same experiments were carried out using a pre-trained YOLO v2 model. The YOLO counterpart gave an accuracy beyond 97% in the absence and in the presence of the intruder. In other words, this work slightly outperformed the YOLO counterpart in terms of the accuracy in both cases, which indicates the robustness of this work. However, it took the YOLO counterpart an hour or so to locate a rat contained in the frames, which highlights the contribution of this work.

## Introduction

In the field of epidemiology and molecular genetics, rodents have long been used to investigate basic mechanisms of spatial learning and memory^1–4^. Devices and methods for assessing learning and memory functions have been extensively used since their development in the early twentieth century^5^. Among behavioral tests, radial-arm mazes (RAMs) have been widely used to quantify the spatial learning situations of healthy and unhealthy animals^6–9^ due to their convenience and high reproducibility. RAMs reveal features that can be used to identify Alzheimer’s disease^10^ or frailty^11^ associated with spatial learning impairment in animals, and exhibit various advantages when assessing spatial learning improvement and progress. For example, animals’ spatial memory is improved after experiencing enriched environments^12^ or exercise stimulation^13^. RAM assessment methods have also been successfully implemented for humans^14^. Many studies have shown that traumatic brain injury (TBI) is a high risk factor for Alzheimer’s disease^15^. TBI, also referred to as head injury or intracranial injury, is an epidemic that arises from brain damage caused by an external force. Approximately 10 million people suffer from TBI each year^16^. TBI and Alzheimer’s disease share many common pathological features, such as deposits of β-amyloid protein (Aβ) in the brain, neurofibrillary tangles, neuronal synapse loss and cognitive impairment^17^. Therefore, a RAM is used in this work to explore the behavioral characteristics of rodents with TBI.

Tests entail an eight-arm radial maze. Food is placed at the ends of some of the arms, and animals’ spatial memory performance can be quantified as quantities such as the number of times each arm is accessed, the amount of time spend in each arm, the number of times that baited and non-baited arms are accessed, and the sequence of accessed arms^6^. Short-term and long-term memory are evaluated after a maze test. Short-term memory, also referred to as the working memory, is the transient memory of task-related information in competitive environments^18^. By contrast, long-term memory, also referred to as the reference memory, is the permanent memory developed in natural adaptation to repetitive stimulation by the processing of the same information^18^. In addition to the short-term and long-term memory, other quantities related to search behaviors merit further investigation.

Although search trajectories in mazes can serve as an indicator, they are mostly used in the studies related to water mazes^19,20^, while hardly used in RAM-based studies. Some water maze-based studies have suggested that animals with variant pathologies adopt different search trajectories. For example, rats with hippocampal lesions circled around their targets, whereas those with global cerebral ischemia wandered around their targets^21^. However, few studies have been conducted on the search trajectories of animals due to a poor repeatability^21^ in a water maze test, simply because rats escape by swimming under an extremely high level of stress. Therefore, a water maze is not a good choice to objectively assess the memory of animals with motor function impairment. Stress has been acknowledged as a confounding factor to the accuracy of water maze experiments. Instead, there is a higher reproducibility in RAM tests, since test subjects experience relatively low psychological stress when food is used as an incentive in tests. Thus, an RAM is used to investigate the food search trajectory of rats with cognitive impairment.

Position tracking technologies for animals can be broadly categorized into contact and non‐contact. In the former case, tracking data were provided using sensors affixed to an animal^22,23^, while positions were evaluated in the latter case^24,25^. Non‐contact tracking is common in laboratories where software tools calculate animals location from video footage using software tools^24–32^. However, as will be seen below, each tracking strategy has its own advantage and disadvantage.

Background subtraction strategy works well on the condition that the background is static while the animals are mobile^29–31^, and remains one of the commonest computer vision techniques for object detection. It can handle complex backgrounds and take care of non-uniform illumination problems. However, it fails when the tracked animals remain static for a long period of time, or if the illumination varies over time. Adaptive thresholding technique is a simple computer vision technique used to segment an object of interest from its background by binarizing an image^33^. Using the adaptive thresholding technique skillfully, animals can be reliably located from non-uniformly illuminated or non-static backgrounds, e.g. disturbed water^26^. Use of a Kalman filter had been validated as an efficient way to deal with tracking issues^25,34^. A wide variety of tracking algorithms have been proposed as well, e.g. identity matching^24^, optical flow^28^ and model-based tracking^32^.

Recently, deep learning models have been well applied to target tracking issues^35–37^, including automatic detection of marine species in aerial imagery^38^ and identification of individual animals in a crowd^39^. Many high-performance algorithms employ convolutional neural networks (CNNs) as feature extractors. You-Only-Look-Once (YOLO) has been acknowledged as one of the most powerful deep neural network (DNN) models in the field of real-time target tracking^40^. A large number of tracking algorithms have been proposed for different purposes. Open-source tracking software is released to the general public, while nobody but programmers can modify the codes to meet their specific requirements. For this sake, commercial software is the No. 1 choice to track animals in the vast majority of experiments^20,41,42^, e.g. Ethovison XT (Noldus, Netherlands), ANY-maze (Stoelting, USA) and SMART video tracking system (Panlab, USA). Despite a licensed copy of SMART video tracking system^42^ available in our laboratory, our team decided to develop our own tracking algorithm for the following reason.

The droppings of a rat are liable to be misidentified as a forever nonmoving target by the SMART video tracking system, and the worst case scenario is that an experiment goes endless regardless of the rat searching for food. As a straightforward, but neither elegant nor efficient, solution, droppings are mopped up manually and instantly so as to keep the image recognition algorithm working. It would take an experimenter hours to watch all the rats go through experiments. In a light room, the droppings of rats reflect light, and hence become noise sources to signal processing. The noise levels could be lowered in a dimly lit room, and all the experiments herein were performed in darkness accordingly.

Here is another reason for our team to develop this work. In the 2-week training program prior to a radial arm maze test, rats were pre-trained to locate the food in 4 out of 8 arms. Interestingly, some rats seemed well satisfied, and stayed still for as long as 40–50 min sometimes after they were “treated” with food at the end of a baited arm. Hence, an experimenter must be alerted to guide the rat to move on to the next baited arm(s). However, the intrusive experimenter is definitely a high-level noise source to the position tracking algorithm. In other words, a rat must be located in real time for efficient training, and a high robustness against an intruder is also required to avoid misidentification. As will be seen below, the presented position tracking algorithm was designed in such a way that both requirements can be fulfilled.

## Methods

**Figure 1:**
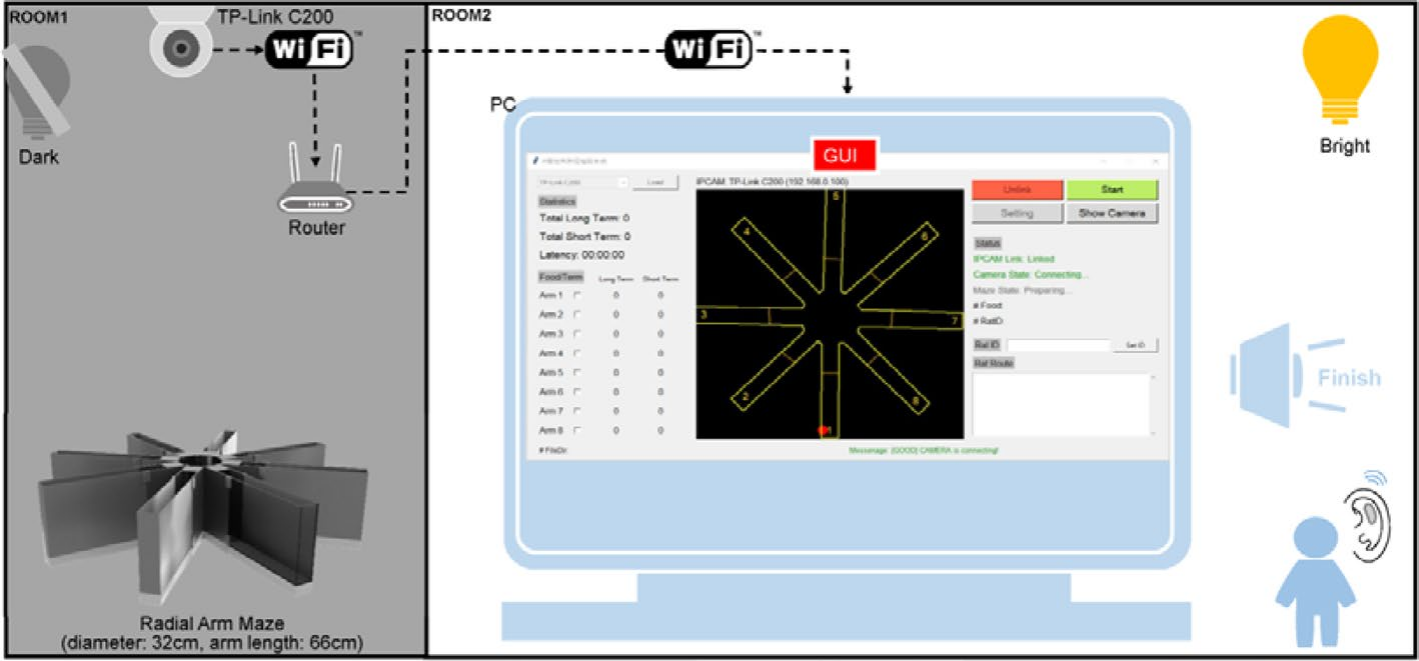
Configuration and framework of the developed system.

This work aimed to develop a real-time tracking algorithm with a high robustness against an intruder by which the food search trajectories of rats can be monitored and then analyzed. An infrared night-vision camera (model: TP-Link C200) was placed 2.2 m above the maze to capture rat images in darkness, as shown in Fig. 1. Experiments were conducted in a dimly lit room, where the camera transmitted real-time images wirelessly to a computer in another room for statistical analysis. An experimenter watched the food searching progress in real time through a user interface (UI). The computer beeped when the searching job was done. Thus, there was no need to the experimenter to remain on standby over the course of an experiment.

### Automatic analysis system

**Figure 2:**
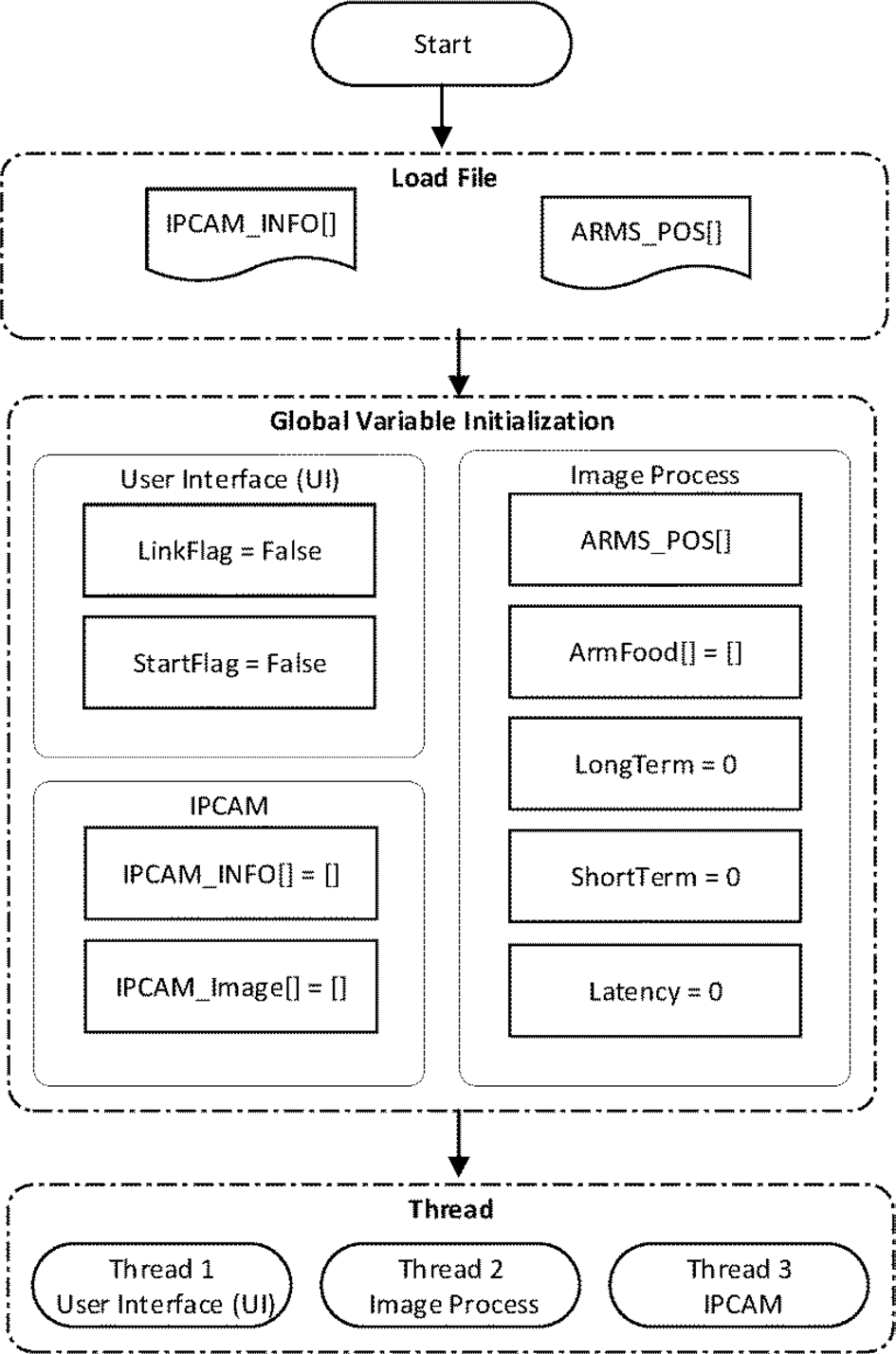
Software flow.

The codes were written using Python. As illustrated in Fig. 2, the flow is divided into three stages: load files, global variable initialization and thread initiation. The “load files” stage comprises the information of a selected camera (IPCAM_INFO[ ]) and the outline of the RAM (ARMS_POS[ ]); the “global variable initialization” stages comprises variables related to the UI, the camera (IPCAM) and image processing; finally the “thread initiation” stage contains three threads, namely the UI thread, IPCAM thread and Image Processing thread.

**Figure 3:**
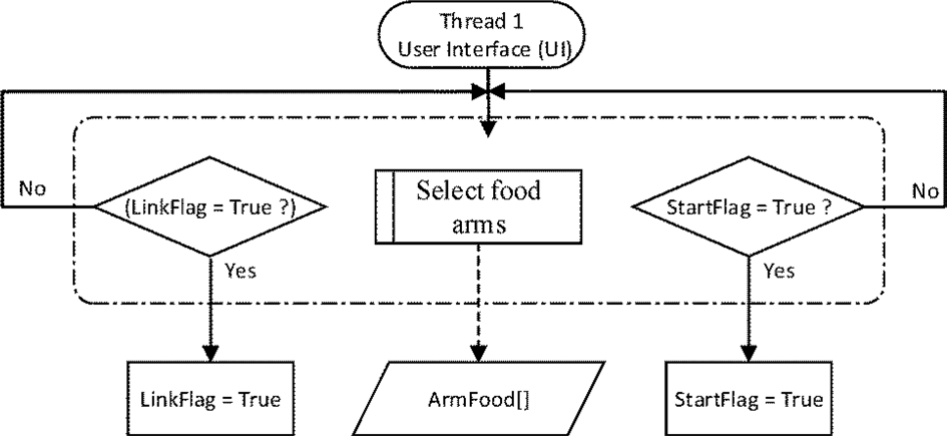
Flow of the UI thread.

As illustrated in Fig. 3, all the baited arms were specified in AF[ ] by an experimenter in the UI thread, and the collected data were used to quantify the cognitive performance of a rat. The UI thread can tell whether the Link or the Start button is clicked to connect the system to the IPCAM or to initiate a system operation, respectively.

**Figure 4:**
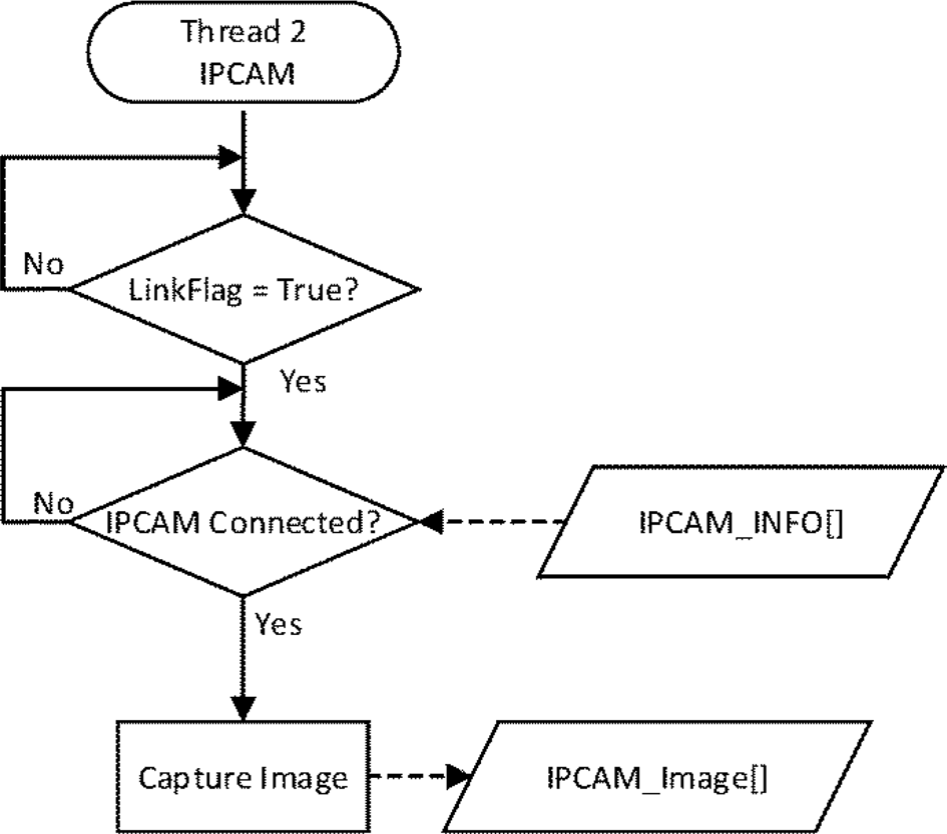
Flow of the IPCAM thread.

**Figure 5:**
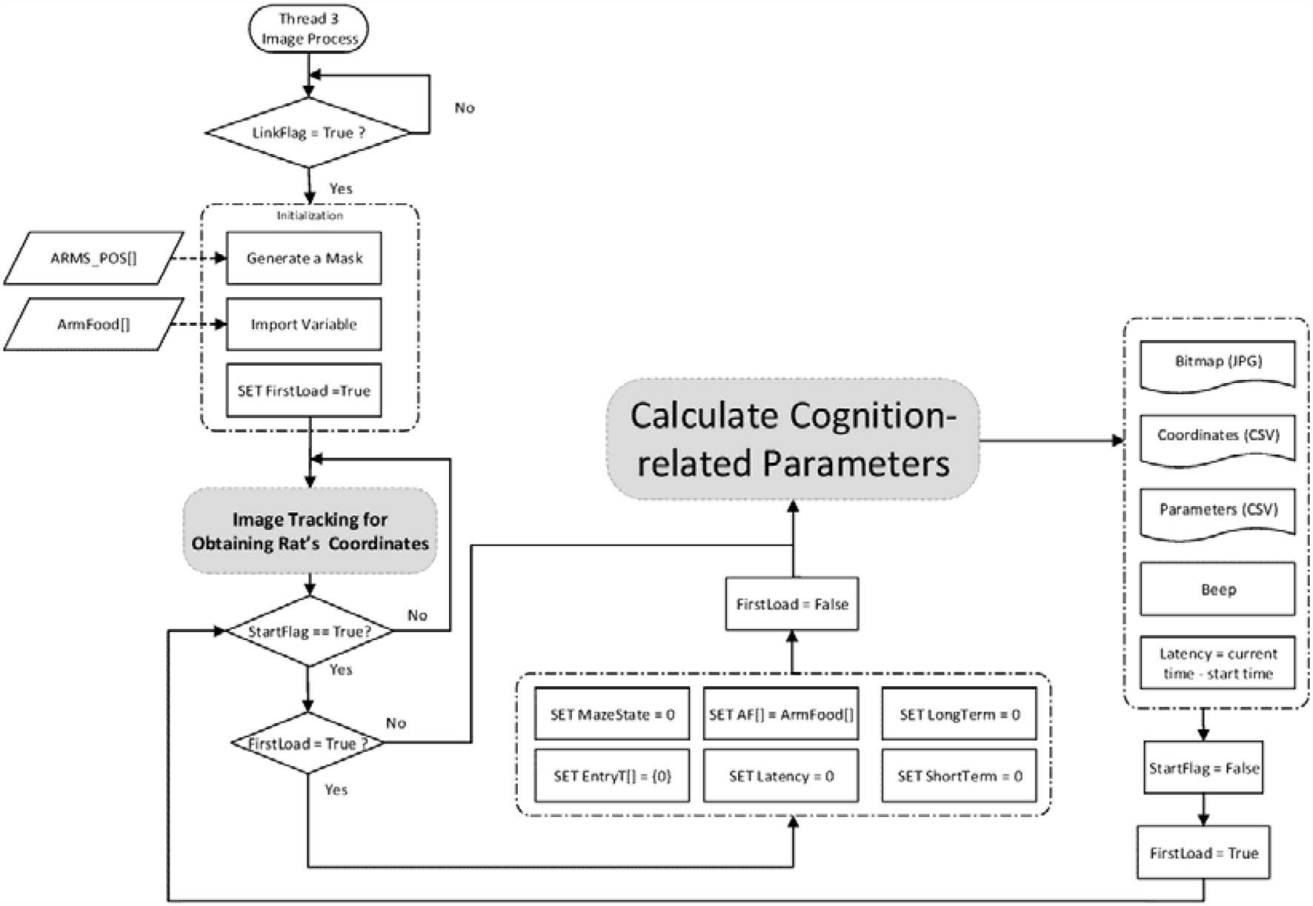
Flow of image processing thread.

The IPCAM thread was designed to tell whether the Link button in the UI was clicked. If it was clicked, “LinkFlag” was set to true and the camera driver information (IPCAM_INFO[ ]) was compared, as illustrated in Fig. 4. Images were captured and saved in IPCAM_Image[ ] after the right camera driver was selected. As illustrated in Fig. 5, the Image Processing thread was designed to locate rats and evaluate cognitive quantities.

**Figure 6:**
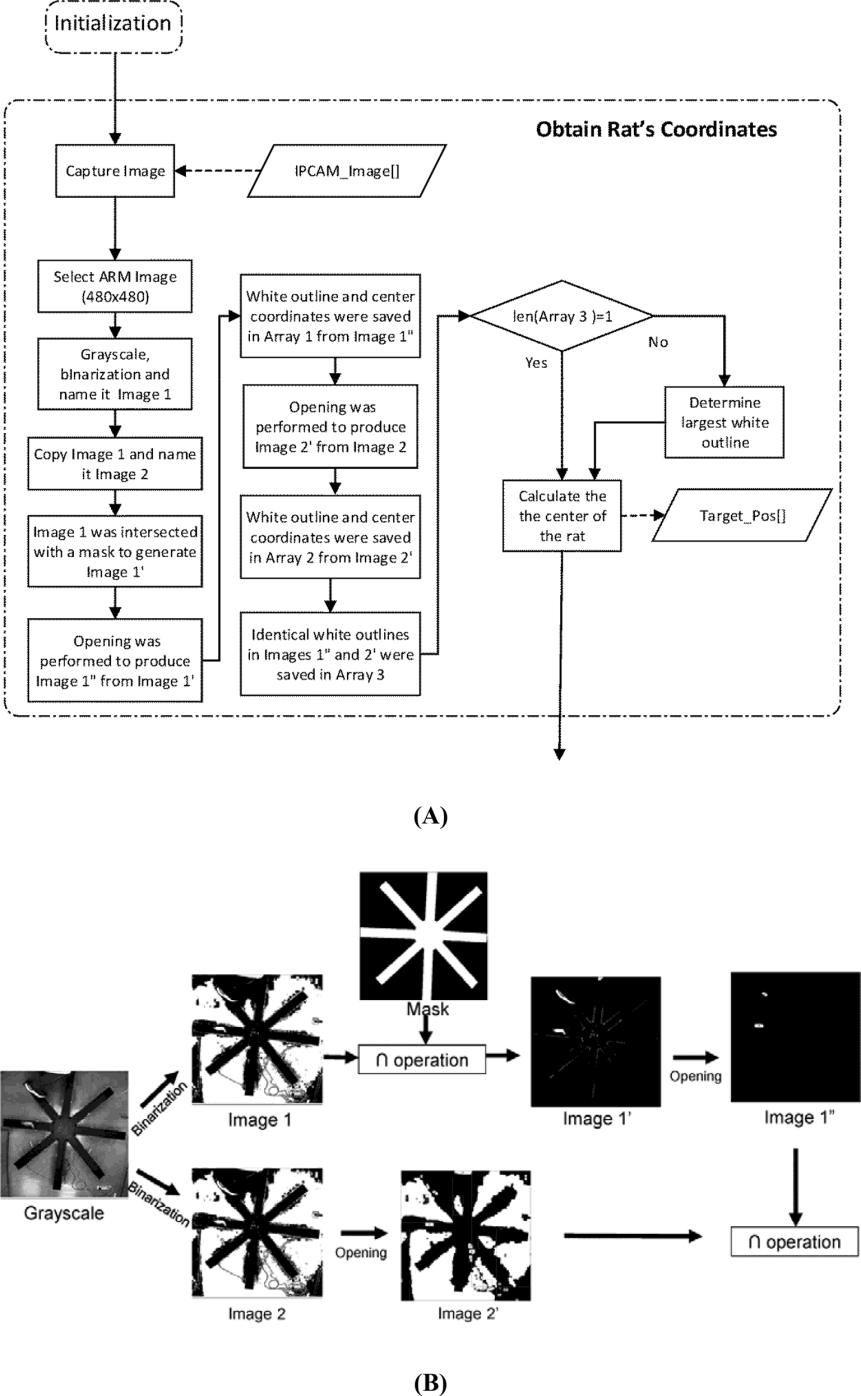

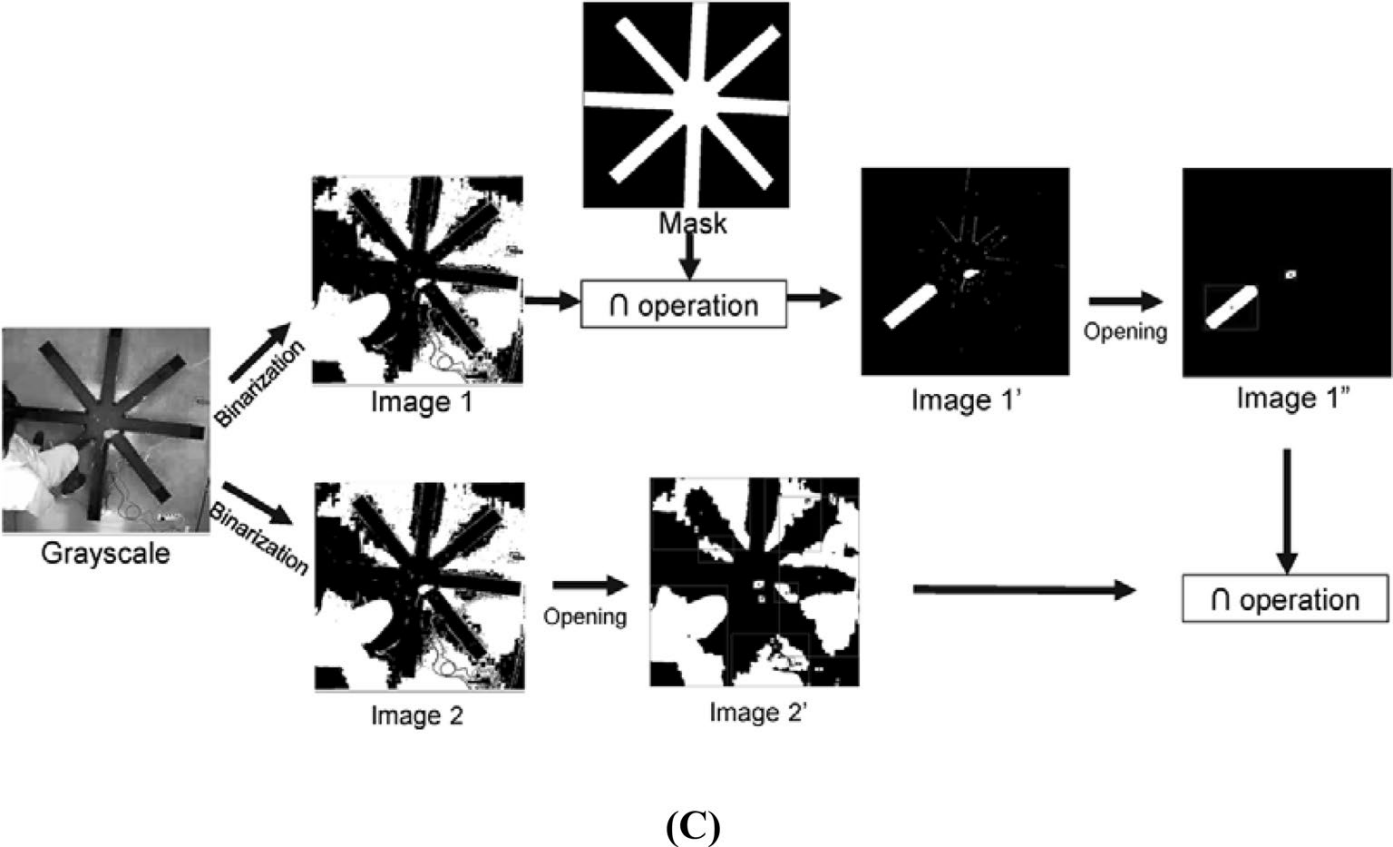
(**A**) Position tracking procedure for rats, (**B**) removal of noise spots caused by an experimenter crossing an arm, and (**C**) by the experimenter whose body covered part of the RAM.

A rat was located as follows. In this work, an animal image was tracked using the OpenCV library^43^. First, an IR image (i.e., IPCAM_Image[ ]) was captured, a subimage of 480 × 480 pixels, containing the RAM, was selected, converted to grayscale and then binarized. The gray level at pixel (i, j) is given by

1 $$\mathrm{Gray}\left(\mathrm{i},\mathrm{j}\right)=0.299\times \mathrm{R}\left(\mathrm{i},\mathrm{j}\right)+0.589\times \mathrm{G}\left(\mathrm{i},\mathrm{j}\right)+0.114\times \mathrm{B}\left(\mathrm{i},\mathrm{j}\right),$$

and lies between 0 and 255. Accordingly, gray levels are binarized with a threshold of 127. The binarized pixel value was set to 1 if Gray(i, j) > 127, and 0 otherwise. A mask used to generate Image 1’ in Fig. 6B, C was simply the outline of the RAM for a subsequent intersection operation. A 5 × 5 matrix is employed as a structuring element for an opening operation. The binary image was duplicated as Images 1 and 2 in Fig. 6. Image 1’ was simply the intersection of Image 1 and the mask in Fig. 6B. Next, an opening operation was performed on Image 1’ to remove the noise spots therein so as to give Image 1″. An opening operation of an image *A* by the structuring element *B* in Eq. (2) is defined as an erosion, expressed in Eq. (3), followed by a dilation operation expressed in Eq. (4). Then, the white outline and the coordinates of the center were saved in Array 1. As before, an opening operation was performed on Image 2 to remove the noise therein so as to give Image 2′. Subsequently, the white outline and the coordinates of the center were saved in Array 2. Finally, the coordinates of both white outlines in Images 1″ and 2′ were saved in Array 3. If Array 3 contained a single white outline, the outline denoted the rat’s body outline. This outline was then used to calculate the coordinates of the center of the rat (Target_Pos[ ]). If multiple white outlines were contained in Array 3, it means that there were noise spots in the images. Here is an example. As depicted in Fig. 6B, the images of a leg and a shoe were captured when an experimenter crossed an arm. The binary image showed white spots and strips caused by the rat and the shoe sole. In this case, the largest white outline inside the maze in Image 1″ was treated as the rat’s outline for the following reason. Most noise spots appeared outside the RAM, and were removed after an opening operation. The residual noise spots after the opening operation were mostly smaller than the rat, and accordingly the largest area contained in Array 3 was simply the rat’s body outline, which was then used to evaluate the coordinates of the center of the rat (Target_Pos[ ]).

Here is another example to illustrate the noise spot removal process. As illustrated in Fig. 6C, the maze was intruded by an experimenter whose body covered part of the maze arms. In this case, binarization and an opening operation were performed to obtain Image 2’. An intersection operation was subsequently performed on Image 2’ and 1’’ to give a white spot as the location of the rat. The coordinates of the center of the rat was saved in a hard drive every 0.05 s.

2 $$ {\text{A}} \circ {\text{B}} = ({\text{A}}{ \ominus }{\text{B}}) \oplus {\text{B}} $$

3 $$ {\text{A}}{ \ominus }{\text{B}} = \left\{ {{\text{z}}\,{|}\,(\hat{B})_{z} \subseteq {\text{A}}} \right\} $$

4 $$ {\text{A}} \oplus {\text{B}} = \left\{ {{\text{z}}\,{|}\,(\hat{B})_{z} \cap {\text{A}} \ne \phi } \right\} $$

**Figure 7:**
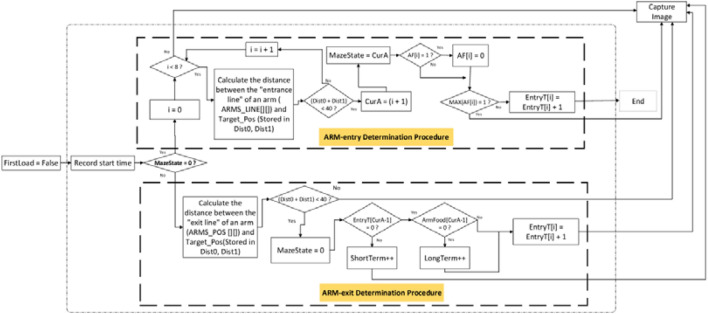
An “access to arms” monitoring procedure.

As illustrated in the upper half of Fig. 7, a variable “MazeState” was used to indicate whether an arm was accessed. MazeState = 0 signified that any arm had not been accessed yet, and then initiate an “access to arms” monitoring procedure. A rat was released in the central platform, and an experiment started. The distance between the proximal end of an arm (i.e., ARMS_LINE[ ][ ]) and the rat’s center (Target_Pos[ ]) was evaluated. If the distance was less than a threshold, the rat was seen as having entered into arm. In this scenario, MazeState was set to the numbering of the accessed arm (MazeState = CurA). Subsequently, another variable AF[i] was used to indicate whether an arm was baited or not. AF stands for Arm Food, and the argument i ranges between 0 and 7. AF[i] = 0 and 1 represent that arm i + 1 is non-baited and baited, respectively. For example, AF[0] = 0 represents arm 1 is non-baited, and AF[1] = 1 represents arm 2 is baited, and so on. If the food in arm i + 1 was taken, AF[i] was reset to 0, and Entry[i] = Entry[i] + 1. Entry[i] represents the number of times that arm i + 1 has been accessed. Finally, another variable MAX(AF[i]) = 1 was used to represent that there was an unaccessed baited arm(s). Otherwise, MAX(AF[i]) = 0 represents that there was no leftover food in the maze, and the RAM test was completed.

MazeState ≠ 0 represents that the rat stayed inside an arm, and then initiate the procedure as illustrated in the lower half of Fig. 7. The distance between the proximal end of an arm (i.e., ARMS_POS [ ][ ]) and the rat’s center (Target_Pos[ ]) was evaluated again. If this distance was less than a threshold, the rat was seen as having exited the arm. In this scenario, MazeState was reset to 0. Entry[i] ≠ 0 represents arm i + 1 had been accessed before, and this time was a reentry thereto. Therefore, short-term memory error was added by 1. Entry[i] = AF[i] = 0 represents that arm i + 1 is a non-baited arm, and the first entry therein caused a long-term memory error, that is, the long-term memory error was added by 1.

### UI of the developed system

**Figure 8:**
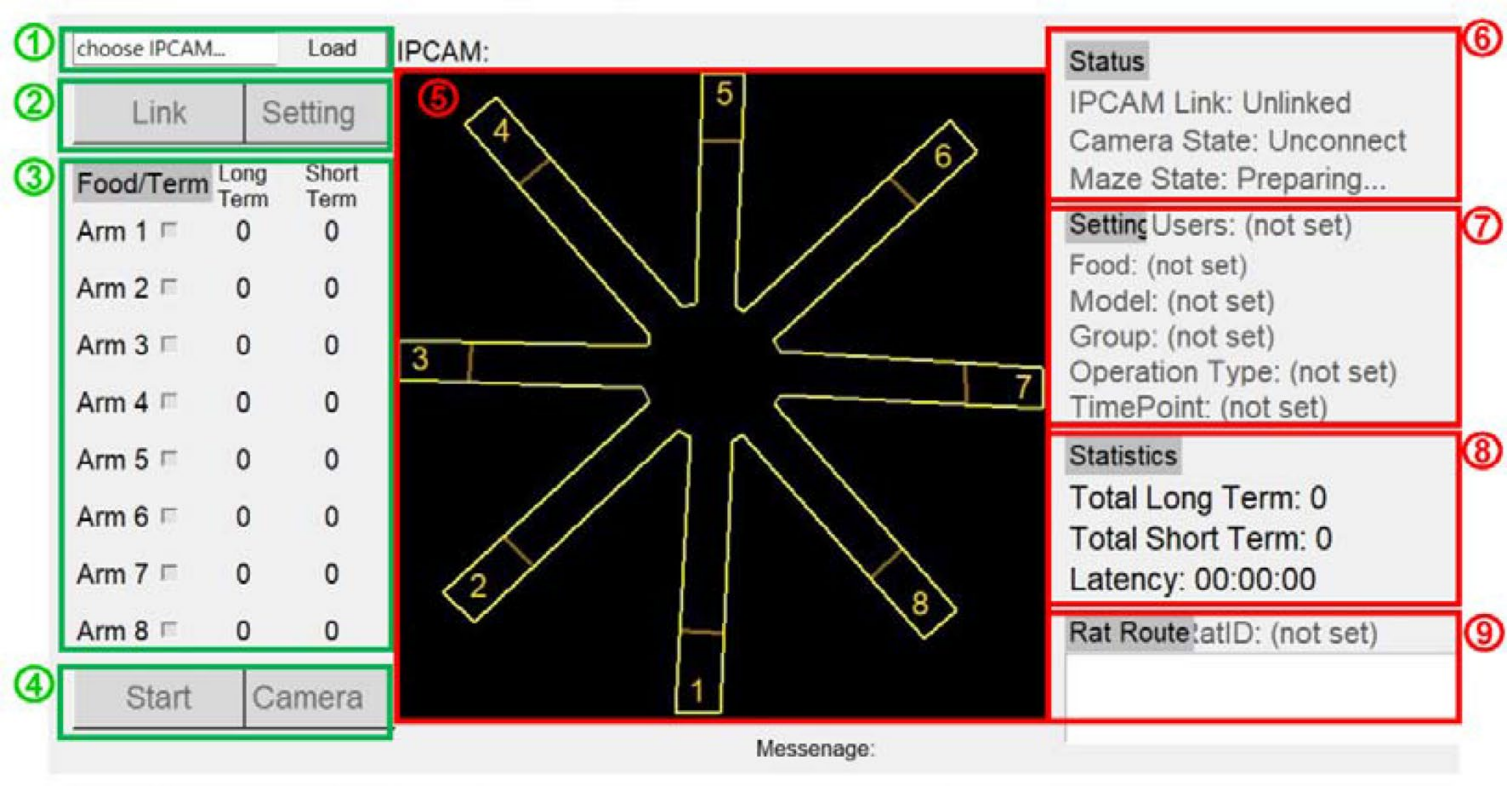
UI of the developed system.

**Figure 9:**
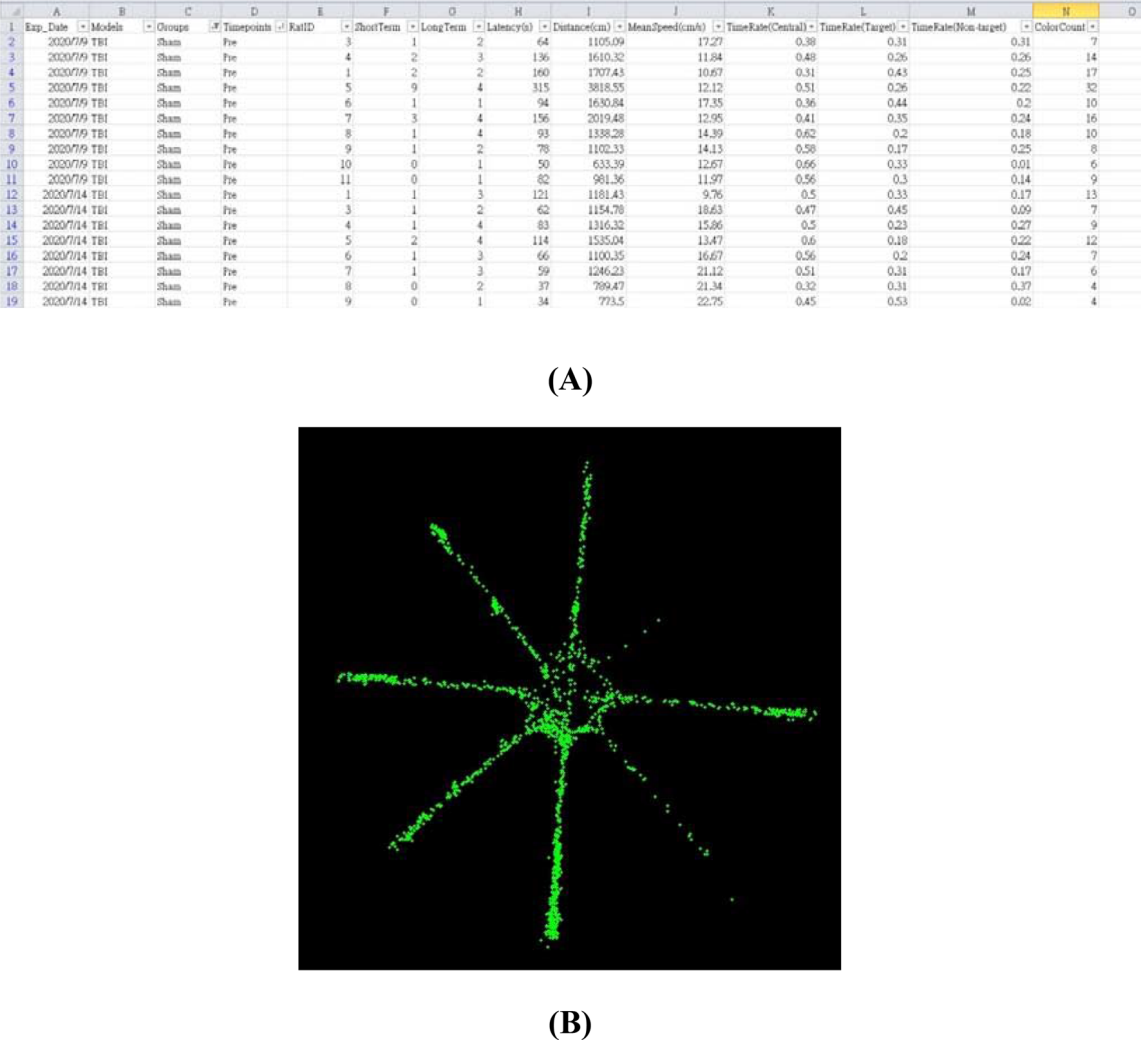
(**A**) cognitive quantity display and (**B**) food search trajectory display.

Figure 8 illustrates the UI of the developed system. Here is how it works. First, the camera was selected, the “Link” button was clicked to connect to the camera, and then the connection status was displayed in Frame 6. Next, the “Setting” button was clicked to specify the information of a rat, e.g., model and group, and the information entered was displayed in Frame 7. Finally, the baited arms were specified and the “Start” button was clicked to enable a system operation. Images of the rat can be seen by clicking “Camera” in Frame 4. In Frame 5, the location of the rat was displayed in real time. The long-term, short-term memory error and the latency were presented in Frame 8, and quantities such as the sequence of accessed arms were shown in Frame 9. After a test was completed, the system automatically terminated, saved all the quantities as an excel file in Fig. 9A and a food search trajectory as a bitmap file in Fig. 9B.

### Experimental procedure for inducing TBI

#### TBI surgery

Animal models with TBI were obtained by inflicting a fluid percussion injury on healthy rats using a fluid percussion device (VCU Biochemical Engineering, Richmond, VA, USA). The percussion force generated was 1.8–2.0 atm, and this force damaged the rats’ brains. Prior to the experiment, the rats were completely anesthetized by introducing a Zoletil mixture into their bodies through intramuscular injection. Next, the rats were tethered to a Kopf stereotaxic instrument, where their heads were fixated by placing an iron rod in each of their two ear sockets. Then, the epidermises of the rats’ heads were cut open, which exposed the bregma and lambda of their skulls. The point 3.0 and 4.0 mm behind the bregma and to the right of the bregma, respectively, was set as the center point of percussion. Subsequently, the rats’ skulls were scraped off using a drill, and an injection cap was fixed to their skulls. The injection cap was connected to the fluid percussion device to induce percussion. For the Sham group, sham surgeries were performed and no percussion force was applied.

#### Experimental protocol

**Figure 10:**
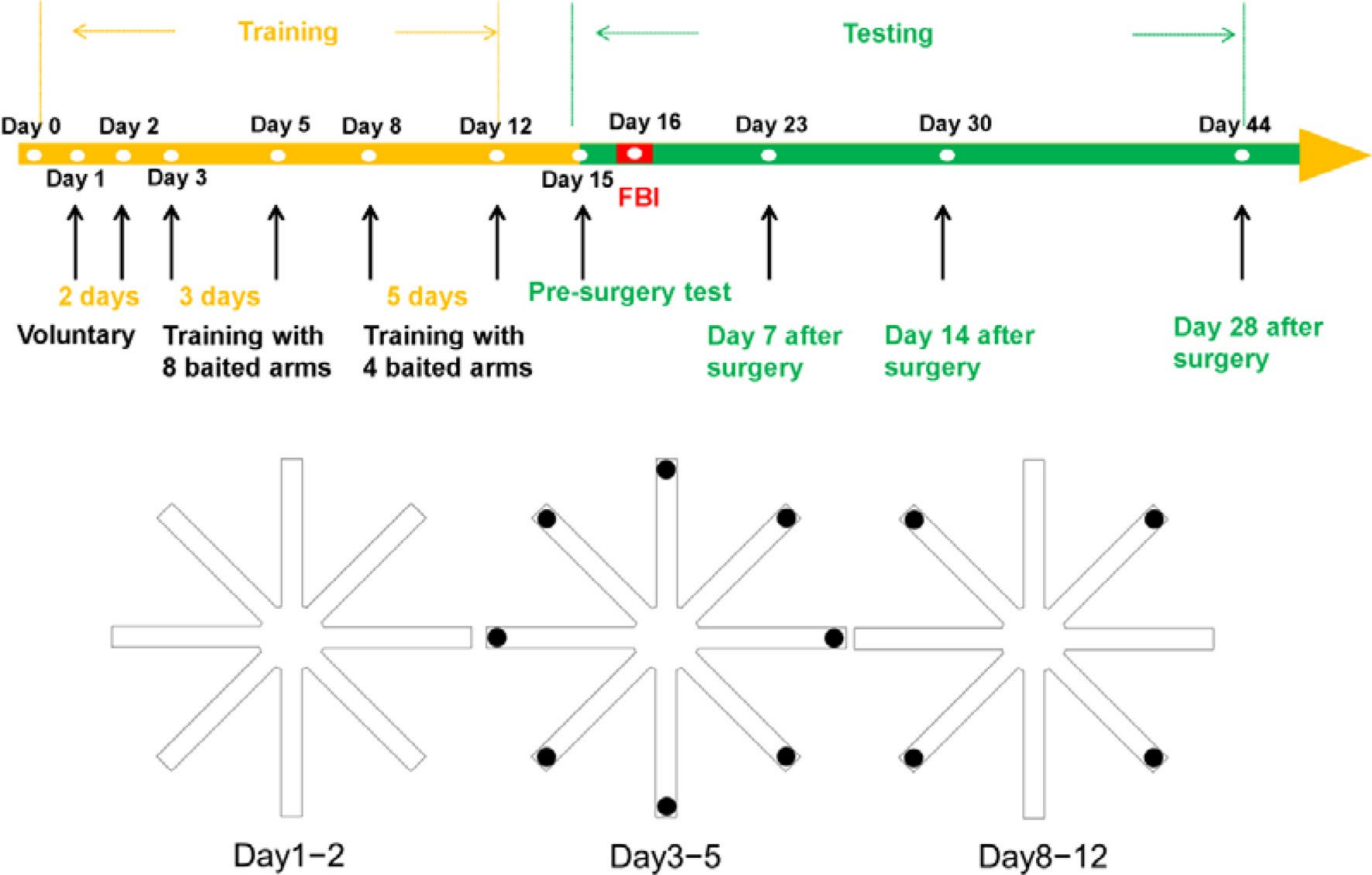
A RAM experimental protocol.

As illustrated in Fig. 10, an experiment was divided into two stages: the training and testing stage. Prior to inducing TBI, all the rats were trained in the RAM for 2 weeks. On Days 1 and 2, the rats were introduced to the maze. No food was placed in any arm to allow the rats to familiarize themselves with the new environment. On Days 3–5, food was placed in the distal end of each arm so as to guide the rats to reach each arm. On Days 8–12, food was placed in the distal end of four arms, and trained the rats to memorize the locations of the four baited arms. A day before the induction of TBI, a presurgery test was conducted in the maze, and the rats took a week of rest afterwards. Then, each rat was subjected to a maze test on a weekly basis with the same baited arms as before. The tests lasted for 1 month. The experimental protocol was approved by the animal ethics committee of Chi Mei Medical Center, Tainan, Taiwan. The study was conducted in compliance with the ARRIVE guidelines^44^. All the methods proposed here were performed in accordance with relevant institutional guidelines and regulations.

### Ethics approval and consent to participate

The statements on Ethics approval and consent to participate in the study are reported in the Methods–Experimental animals section.

### Consent for publication

Not applicable.

### Results

**Figure 11:**
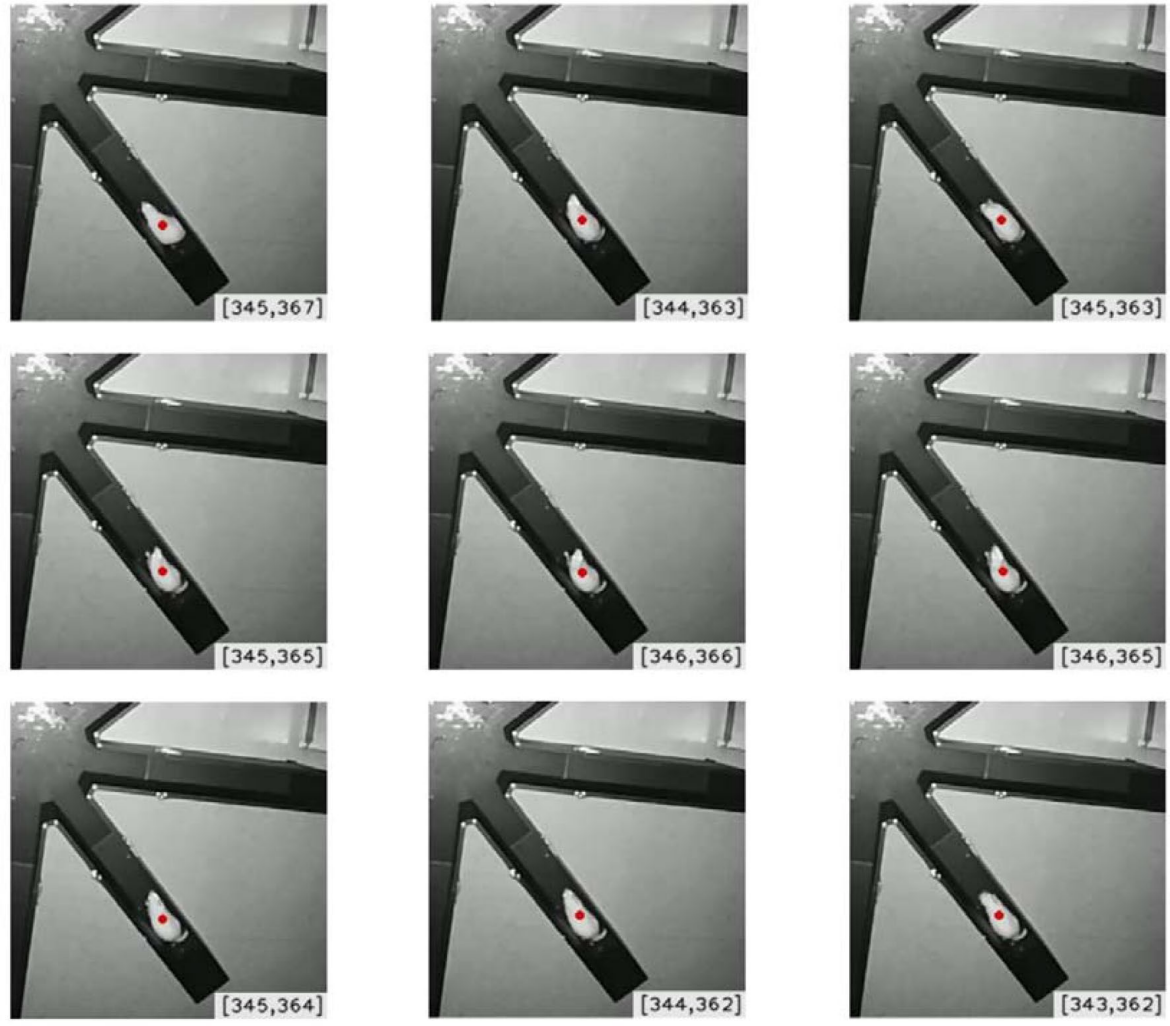
Illustration of an “S” spot regardless of small body movements.

This article aimed to compare the food searching performance, including the search trajectory, between the Sham and the TBI group. However, it was observed that a rat did not keep searching for food in an RAM. For example, as presented in Fig. 11, a rat was detected as a red dot, and regarded as “staying still” if it moved within a specified range. In this fashion, the cognitive function of a rat can be quantified as the total number of spots and the total amount of time that the rat stayed still, which will be seen in the last two rows of Table 1. For the sake of discussion, a red spot, as illustrated in Fig. 11, is referred to as an “S” spot, and the total amount of time that a rat stayed still is referred to as the duration at the S spots hereafter.

**Table 1:** Statistics on cognitive performance.

Cognitive quantities	Timing	Sham (n = 6)	TBI (n = 6)
Latency (s)	Pre-surgery	156.5 ± 73.0	166.5 ± 71.0
	Day 7 after surgery	158.8 ± 104.6	499.5 ± 121.6*
	Day 28 after surgery	181.4 ± 67.9	998.1 ± 291.8*
Short-term memory error (times)	Pre-surgery	1.4 ± 1.3	1.3 ± 1.2
	Day 7 after surgery	1.5 ± 1.4	8.4 ± 3.7*
	Day 28 after surgery	2.1 ± 1.9	18.0 ± 6.1*
Long-term memory error (times)	Pre-surgery	1.5 ± 0.5	1.6 ± 0.6
	Day 7 after surgery	1.8 ± 1.0	3.7 ± 0.5*
	Day 28 after surgery	2.8 ± 0.8	3.5 ± 0.5*
Length of food search trajectory (m)	Pre-surgery	19.2 ± 6.2	18.8 ± 7.5
	Day 7 after surgery	20.9 ± 5.0	35.1 ± 12.1
	Day 28 after surgery	21.9 ± 7.1	56.1 ± 21.5*
Average speed (cm/s)	Pre-surgery	13.2 ± 1.4	12.4 ± 1.4
	Day 7 after surgery	12.7 ± 1.5	8.1 ± 1.1*
	Day 28 after surgery	14.1 ± 2.5	5.7 ± 1.6*
The time spent in the central platform (%)	Pre-surgery	60.5 ± 22.4	51.6 ± 20.1
	Day 7 after surgery	55.1 ± 9.8	41.3 ± 11.3
	Day 28 after surgery	63.6 ± 11.8	28.8 ± 5.6*
The time spent in the baited arms (%)	Pre-surgery	12.7 ± 5.5	13.2 ± 4.4
	Day 7 after surgery	11.8 ± 3.9	39.0 ± 16.2*
	Day 28 after surgery	16.1 ± 3.4	40.8 ± 11.6*
The time spent in the non-baited food arms (%)	Pre-surgery	24.7 ± 5.1	33.0 ± 12.4
	Day 7 after surgery	29.7 ± 5.4	30.0 ± 8.6
	Day 28 after surgery	30.7 ± 7.9	31.7 ± 15.6
The number of the S points	Pre-surgery	38.6 ± 25.8	40.3 ± 20.7
	Day 7 after surgery	48.6 ± 16.2	123.1 ± 32.4*
	Day 28 after surgery	52.75 ± 11.3	266.5 ± 58.7*
Duration at the S points	Pre-surgery	52.2 ± 20.1	54.2 ± 24.0
	Day 7 after surgery	55.5 ± 24.3	290.0 ± 102.2*
	Day 28 after surgery	67.6 ± 17.6	660.8 ± 188.3*

Note that values are expressed as mean ± S.D. A student’s t-test was used to compare performance between both groups. *p < 0.05 indicated a statistical significance as compared with the sham group.

**Figure 12:**
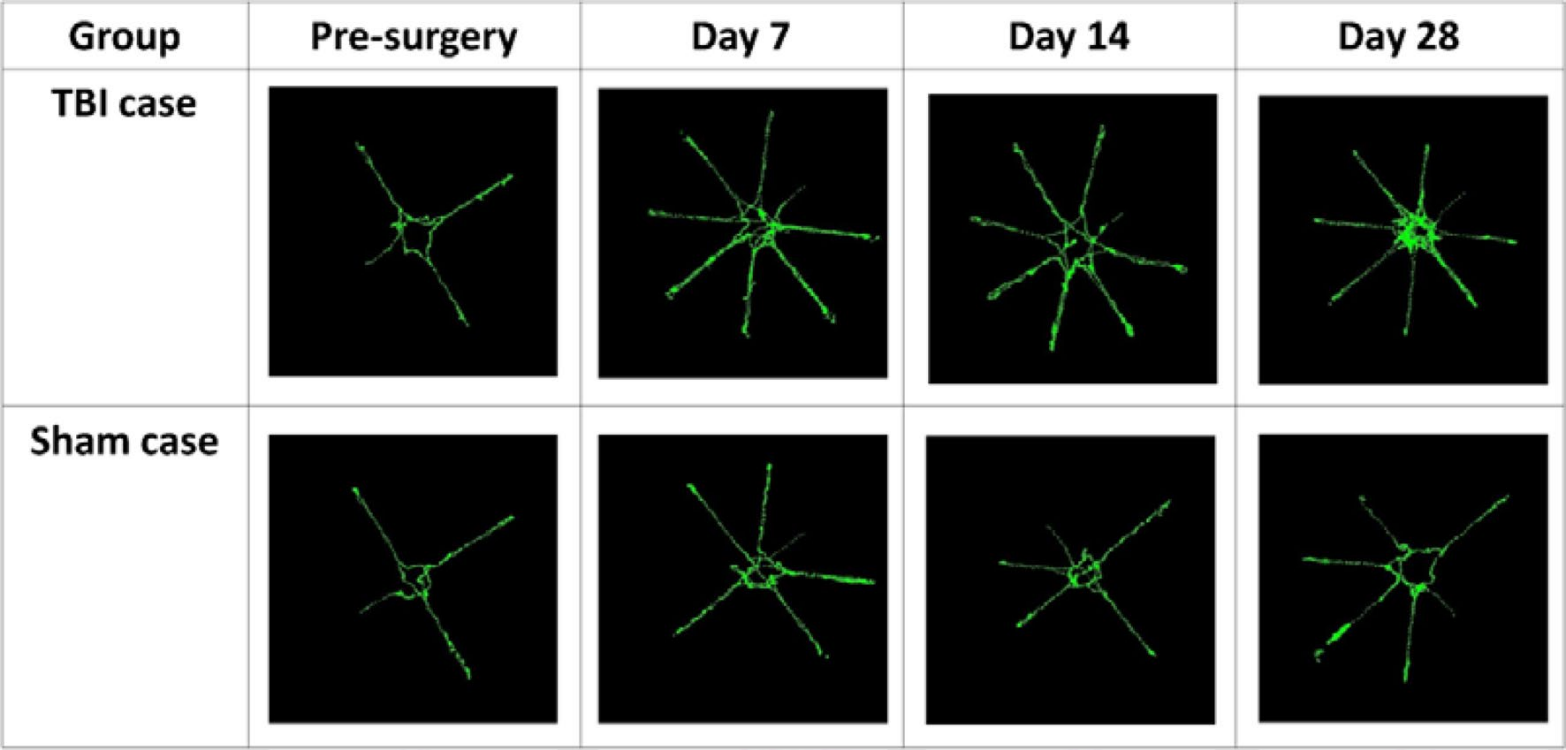
Food search trajectory comparison between the TBI and sham group over the course of 1 month.

Figure 12 presents a comparison on the food search trajectory between both groups over the course of 1 month. As time went by, a rat in the TBI group accessed more arms, eventually all the baited and non-baited arms, for food. Besides, it had more repeated access to the arms than earlier. Instead, a rat in the Sham group had access to less arms than in the TBI case, meaning that the rat had a better spatial memory than the TBI counterpart.

**Figure 13:**
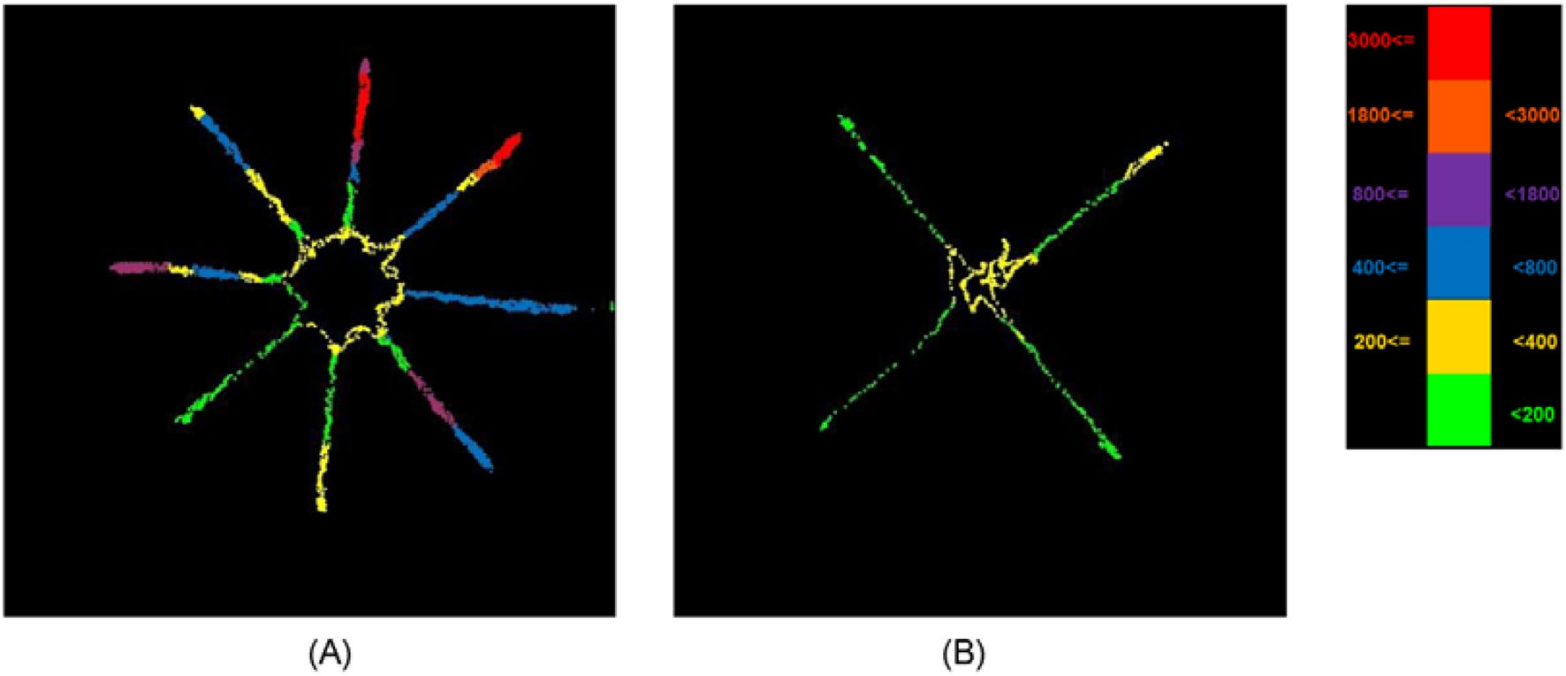
Dot distribution over the entire maze of another single rat in (**A**) the TBI group and (**B**) in the Sham group on Day 28.

Each arm of the maze is 70 cm long and 10 cm wide, and was equally divided into 10 pieces for statistical purpose, each of which occupied an area of 70 cm^2^. In addition, the central platform of the maze was divided into 9 pieces, each with an area of 70 cm^2^ approximately. Therefore, the maze was divided into a total of 89 pieces. A rat was detected as a dot which was displayed in color according to the number of times that the rat stayed in a piece, as in rainfall statistics. For illustrative purposes, the food searching pattern of another rat in the TBI group was presented in Fig. 13A. Readers may be misled into thinking that the rat kept accessing all the arms, but did not reach the central platform of the maze. However, the food searching patterns vary among individuals, and this observation cannot apply to others in the TBI group. For instance, two rats in the TBI group kept circling along the rim of the central platform, but hardly entered an arm. Besides, there are more dots in Fig. 13A than in Fig. 13B, simply due to a longer food search trajectory required in the TBI group than in the Sham counterpart on Day 28, as will be seen in Table 1. It is also noted that there are denser dots in the distal than the proximal end of some arms. This finding could be ascribed to the fact that food was placed in the distal end of the baited arms.

Table 1 presents a performance comparison between the TBI and the sham group over 28 days. There were significant difference in the latency, short-term and long-term memory error on Day 7, and the performance gap widened on Day 28. The length of a food search trajectory is defined as the sum of all the distance between two consecutive detected dots. There were significant difference in the length of a food search trajectory and the average speed between both groups on Day 28. Over time, the TBI group took a longer route and at a slower average speed to complete tests. Moreover, the proportion of time spent by the TBI group in the central platform decreased over time, whereas that by the sham group was maintained at 50–60%.

Over time, the proportion of time spent in the baited arms increased in the TBI group, and hit approximately 40% on Day 28. In contrast, it lay between 10–20% in the Sham group, which was statistically distinguishable from 40% in the TBI group. There was statistical difference in the quantity between the two groups on Days 7 and 28. Instead, the time spent in the non-baited arms in both groups reached approximately 30%, and there was no statistical difference between both.

Finally, the last two rows indicate that there were 40.3 ± 20.7 S spots before TBI surgery, while at 123.1 ± 32.4 S spots seven days later in the TBI case. Likewise, the duration at the S spots was raised from 54.2 ± 24.0 s before TBI surgery to 290.0 ± 102.2 s seven days later. All the figures were statistically distinguishable from those in the Sham case, and the performance gap between both groups grew over time, meaning that the cognitive function of the TBI group worsened over time.

On the issue of robustness, the accuracy and the tracking time were measured in two cases to compare the performance between this work and a You Only Look Once (YOLO) v2 model. The accuracy was measured as the detection rate which is defined as the percentage of the frames in which a rat was reliably detected, and the tracking time is defined as the amount of time taken by an algorithm to track the rat throughout the frames. The YOLO v2 model was pre-trained using 100 labeled images of rats captured in the RAM, and then tested on 9010 frames extracted from a 5-min video in Cases 1 and 2. The only difference between Cases 1–2 is simply that the 5-min video in Case 2 contained an intruder into the RAM, while that in Case 1 did not. Algorithms were executed on an Altos BrainSphere™ P550 F4 Workstation running Windows 10 Enterprise edition, equipped with dual Intel(R) Xeon(R) Gold 5220R 2.20 GHz processors and 16 GB of RAM. Table 2 gives a performance comparison between the YOLO v2 model and this proposal. As it turned out, this work slightly outperformed the YOLO v2 counterpart in terms of the detection rate in both cases. It is also noted that the detection rate in Case 2 is slightly below that in Case 1 (97.45% vs. 97.75% in YOLO v2, and 98.19% vs. 99.10% in this work), meaning that both strategies have a high robustness against an intruder. Here, it must be pointed out that this work is far more efficient than the YOLO v2 counterpart, that is, 3 vs. 50 min in Case 1, and 2 vs. 73 min in Case 2. As referenced in the Introduction, the real-time position tracking feature is the key to this work, and this feature gives the presented algorithm a clear advantage over the YOLO v2 model.

**Table 2:** Performance comparison between this proposal and a YOLO v2 model.

Video	Tracking time (h:min:s)		Detection rate (%)	
	YOLO v2	The study	YOLO v2	The study
Case 1	00:50:28	00:03:43	97.75	99.10
Case 2	01:13:23	00:02:33	97.45	98.19

## Discussion

In agreement with others’ work in the literature, the latency, short-term and long-term memory error grew over time in the TBI group. In this work, it was particularly seen that two newly introduced quantities, the number and the duration at the S spots, were raised over time in the TBI group, meaning that both quantities can be viewed as measures of cognitive impairment. It was also observed that another two newly introduced quantities, i.e. the length of food search trajectory and the average speed, increased and decreased over time. Besides, another interesting finding is that the TBI group showed different food searching patterns from the Sham group, and the pattern even varied among individuals in the TBI group. Analysis on food searching patterns is definitely an issue that deserves further investigation.

YOLO has been acknowledged as a robust object recognition model if adequately trained. As listed in Table 2, this work provides a comparable detection rate as compared with a YOLO v2 model in Cases 1–2, meaning that it is robust against noise spots formed by the droppings of rats and against a high-level noise source such as an intruder into a maze.

On the other hand, this work significantly outperforms the counterpart in terms of tracking time due to simple operations. The real-time feature gives this work a clear advantage over the YOLO v2 model, and is the key to efficient rat training prior to RAM tests. The real-time advantage of this work is expected to be further highlighted when it is applied to locate a mouse inside a maze more complicated than a RAM^45,46^, for it would take a YOLO model a much longer period of time to locate a target from a complicated background in a frame.

## Conclusion

This paper presents a novel position tracking algorithm by which a rat in a radial arm maze can be located in real time using simple intersection and opening operations. It provides an accuracy above 99%, and also has a high robustness against the noise spots formed by the droppings of the rat. The accuracy slightly fell to 98% when the maze was intruded by an experimenter. As opposed to a YOLO v2 model, the algorithm does not require pre-training. Even though the YOLO counterpart provides comparable accuracy and robustness, it took a much longer period of time to locate the rat in the maze. In other words, the real-time feature gives the work a clear advantage over the YOLO counterpart.

## Data Availability

The data that support the findings of this study are available from the corresponding author upon reasonable request.
